# Comparative Analysis of the Pre-Parturition and Post-Parturition Genital Tract Microbiota in Plateau Bangor Sewa Sheep

**DOI:** 10.3390/vetsci10080523

**Published:** 2023-08-14

**Authors:** Hongcai Ma, Wangsheng Zhao, Tianzeng Song, Zhaxi Baijiu, Zhenzhen Zhang

**Affiliations:** 1 Institute of Animal Husbandry and Veterinary Medicine, Tibet Autonomous Region Academy of Agriculture and Animal Science, Lhasa 850009, China; xzmahongcai@126.com (H.M.); songtianzeng123@sina.com (T.S.); 2 School of Life Science and Engineering, Southwest University of Science and Technology, Mianyang 621010, China; wangshengzhao01@163.com; 3 Cultural Service Center of Maqian Township, Nagqu 852599, China; 18798948899@163.com

**Keywords:** Bangor Sewa sheep, microbiota, genital tract, pre-parturition, post-parturition, plateau

## Abstract

**Simple Summary:**

Microbiota is closely related to animal health. Bangor Sewa sheep are an important biological resource that live on the Tibetan Plateau. In this paper, we compared the microbial composition and structure in the birth canal of Bangor Sewa sheep before and after delivery. A total of three phyla and 74 genera, including a variety of positive and negative health-associated factors, showed significant differences between pre- and post-parturition groups. This research offers a new approach to improving the well-being and productivity of the Bangor Sewa sheep.

**Abstract:**

(1) Background: Bangor Sewa sheep are an economically significant livestock species on the plateau. The roles of microbiota in reproduction are complex and critical for animal health. But little is known currently about the microbiome of plateau Bangor Sewa sheep. The purpose of this study was to discover the changes in the genital tract microbiota of pre- and post-partum Bangor Sewa sheep. (2) Methods: Samples from the birth canal were obtained for 16S rRNA sequencing, three days before and after delivery, respectively. (3) Results: The results showed that there was a noticeable difference in three phyla and 74 genera between the pre- and post-parturition groups in the microbiota of Bangor Sewa sheep. The changes included a decrease in the abundance of genera related to health (unclassified_*Cellulomonadaceae*, *Cellulomonas*, *Fibrobacti*, *Flavobacterium*, *Eubacterium_ventriosum*_group, *Acetitomaculum*, *Aeromicrobium*, *Dietzia*, *Romboutsia*, *Ruminococcus*, etc.) and an increased abundance of negatively related genera (*Nocardioides*, unclassified_*Clostridia*, *Sphingobacteriaceae*, unclassified_*Ruminococcaceae*, *Prevotellaceae*_UCG_004, *Micromonospora*, *Streptococcus*, *Facklamia*, *Bosea*, etc.) spp. (4) Conclusions: Microbes can serve as indicators of the physical state of Bangor Sewa sheep. These findings laid the foundation for deciphering the effects of microbial changes during birth on the reproductive health of plateau Bangor Sewa sheep.

## 1. Introduction

Meat is a beneficial part of people’s diets that provides necessary nutrients such as proteins, lipids, vitamins, and others [1]. Sheep are valuable food animals that provide meat, milk, and other products [2]. Statistically, China possessed over 300 million sheep herds, which supplied 5.2 million tons of mutton and 14.6 thousand tons of cashmere in recent years (we refer the reader to https://data.stats.gov.cn/easyquery.htm?cn=C01 (accessed on 25 May 2023)), both of which had a substantial influence on national life (Figure S1). Bangor Sewa sheep are a native species found in around 4800 m plateau areas of Tibet’s Bangor, Amdo, Dangxiong, Nima, Shenzha, and Shuanghu counties, with a total population of 480 thousand in China. As with yaks and pigs in Tibet, Bangor Sewa sheep are an economically significant livestock species on the plateau. They are known for rapid development, a robust physique, feeding resistance, high milk yield, strong disease resistance, and high stress tolerance in harsh, cold, and high-altitude environments. Due to its distinctive meat quality and nutrient-dense meat, a growing number of consumers are choosing Bangor Sewa sheep products. However, Bangor Sewa sheep are currently facing a serious problem of low reproductive rates, which seriously restricts the development of the industry. It is crucial to protect their health and reproduction for ranchers and the local economy.

The microbiome is made up of billions of bacteria that dwell within, or on the outside, of the host. These microbes significantly affect the host’s health and susceptibility to illness [3]. The microbiome is well-known for its vital functions in the host’s energy metabolism, epithelial health and gut barrier maintenance, immunologic function, and neuron development [4,5,6]. Recently, increasing attention has been paid to exploring the effect of microbiota on animal reproduction [7]. Studies have indicated that the roles of microbiome in animal fertility are multifaceted and crucial for the health of animals. On the one hand, the microbiota in the gut has a symbiotic relationship with animals. They aid in digestion and nutritional absorption, which are essential for the health and well-being of animals [8]. Certain gut microbiota can also influence hormone regulation in animals [9]. These hormones are vital for controlling various aspects of reproduction, including sexual development, estrous cycles, and fertility [10]. In addition, the microbiota in reproductive tracts helps maintain the pH balance and prevent the overgrowth of harmful bacteria, which can prevent infection and promote fertility [11]. On the other hand, microbes can compete for resources and prevent the formation of prospective pathogens, acting as a defensive mechanism against harmful pathogens and lowering the risk of infection and sickness, both of which have a detrimental effect on fertility [12,13].

Compared with the gut microbiota, there are few studies on the microbiome in the genital tract. The microecology of the reproductive tract is made up of microbes, physiological structures, and endocrine factors. The unique environment, including the shedding of vaginal epithelial cells and endometrium, offers favorable conditions for microbial colonization [14]. During calving, microbes prevalent in the environment infiltrate the ewe’s uterus. Pathogenic bacteria will colonize the epithelial mucosa of the vagina and uterus, causing further harm to the body’s health and reproductive potential [15]. The genital microbiome diversity is affected by many factors, some of which are specific to the female such as estrous cyclicity [16] and pregnancy [17]. During the perinatal period, which includes both pre- and post-partum stages, there are significant fluctuations in hormone levels, such as estrogen and progesterone, that can impact microbial composition [18]. Hormonal shifts during pregnancy and labor can create an environment that favors the growth of certain bacterial species, leading to changes in the microbiota [19]. For example, a decrease in estrogen levels after delivery can result in a decrease in Lactobacillus bacteria [20], which are commonly found in the vaginal microbiota of healthy women. The immune system undergoes changes during pregnancy to not only prevent the rejection of the fetus but also to maintain a balance with the resident microbiota. These immune adaptations can influence the composition and diversity of the reproductive tract microbiota [21]. Physiological changes that occur during pregnancy and labor can also impact the reproductive tract microbiota. These changes include alterations to pH, nutrient availability, and tissue structure [19]. For instance, the release of amniotic fluid during childbirth can introduce new microorganisms into the reproductive tract, potentially altering the microbiome composition [22]. Therefore, investigating the microbiological changes in the reproductive tract before and after delivery is essential for unraveling the intricate relationship between the microbiota and reproductive health and has the potential to improve outcomes for both mothers and their newborns.

However, there is little information available on the microbiome in the genital tract of Bangor Sewa sheep. In order to better understand the dynamics and significance of the microbiota during peripartum, the current study was conducted to analyze and uncover the diversity of the genital tract microbiota during the pre-parturition and post-parturition periods in Bangor Sewa sheep.

## 2. Materials and Methods

### 2.1. Sampling

Swab samples were collected from a herd of approximately 300 free-range Bangor Sewa sheep kept by a local herdsman’s family in Bangor County, Tibet, China. Approval for sampling and publication from the farm owner was obtained. All natural mating Bangor Sewa sheep were heat-synchronized, and 10 of them at 5 years old with the same production times and close weights and pregnancy periods were employed in this study. A total of 10 swab samples were collected from the birth canal 3 days before delivery (pre-parturition group, *n* = 10) and 3 days after delivery (post-parturition group, *n* = 10). The samples were collected from the same sheep. Then, the swab samples were placed in sterile collection tubes, properly labeled, and transported to the laboratory at 4 °C for DNA extraction. Animal handling protocols were also conducted following the guidelines and regulations approved by the Animal Ethics Committee of Tibet Autonomous Region Academy of Agriculture and Animal Science and Southwest University of Science and Technology.

### 2.2. DNA Extraction

The MolPure^®^ Stool DNA Kit (Shanghai Yeasen Biotechnology Co., Ltd., Shanghai, China) was used to extract DNA from swab samples according to the manufacturer’s specifications. The concentration of extracted DNA was determined using the Qubit 4 Fluorometer (Invitrogen, CA, USA).

### 2.3. The Genital Tract Microbiome Amplicon Sequencing

The V3-V4 region of the 16S rRNA gene was amplified from the DNA of Bangor Sewa sheep samples using the primer pairs 338F/806R (F: 5′-ACTCCTACGGGAGGCAGCA-3′ and R: 5′-GGACTACHVGGGTWTCTAAT-3′). The PCR reaction was performed in a 10 μL mixture of KOD FX Neo Buffer 5 μL, DNA sample 5–50 ng, 338F (10 μM) 0.5 μL, 806R (10 μM) 0.5 μL, and ddH_2_O up to 10 μL. The PCR procedure includes a 5 min initial denaturation at 95 °C, 30 cycles of amplification (denaturation at 95 °C for 30 s, annealing at 50 °C for 30 s, and extension at 72 °C for 40 s), and a 10 min final extension at 72 °C. The amplicons were then purified and quantified using a PureLink™ Microbiome DNA Purification Kit and a Qubit 4 Fluorometer form Invitrogen, USA. The Hieff NGS^®^ Fast Tagment DNA Library Prep Kit (Shanghai Yeasen Biotechnology Co., Ltd., Shanghai, China) was then used for library construction and sequencing on an Illumina novaseq 6000 (Illumina, San Diego, CA, USA).

### 2.4. Bioinformatic Analysis

The following procedures were used to conduct the bioinformatics analysis. First, the raw data were filtered with Trimmomatic, and the primer sequences were deleted utilizing Cutadapt. The filtered reads were then assembled via USEARCH, and chimeras were eliminated using UCHIME [23]. Second, all of the effective reads from each sample were clustered into operational taxonomic units (OTUs) based on a 97% sequence similarity by employing USEARCH, and OTU taxonomy annotation was performed via Naive Bayes classifier in QIIME2. Third, we rarified the OTU to several metrics, including curves of rarefaction, Shannon index, rank abundance, and species accumulation, and also calculated indexes of Chao1, Ace, Shannon, Simpson, Chao1, and Ace using QIIME2 to reveal the species richness and diversity of microbial samples from the Bangor Sewa sheep. For beta-diversity analysis, the heatmaps of RDA-identified key OTUs, principal component analysis (PCA), unweighted pair group method with arithmetic mean (UPGMA), and nonmetric multidimensional scaling (NMDS) were used to evaluate the similarities between microbial communities of the two groups with QIIME2. Fourth, linear discriminant analysis effect size (LEfSe), Metastats, and Random Forest were used for the quantitative analysis of biomarkers among each group for investigating the statistical significance of the taxonomic difference. Finally, the functional prediction of microbes was performed using PICRUSt2, bugbase, Tax4fun, and FAPROTAX.

### 2.5. Statistical Analysis

Data were presented as means ± standard error of the mean (SEM). The data were analyzed using IBM SPSS (26.0). The unpaired Student’s *t*-test was performed to explore the difference in the proportions of the bacterial compositions and predicted function. Statistical significance was determined by *p* < 0.05.

## 3. Results

### 3.1. Composition Analysis of Microbiota in the Birth Canal of Bangor Sewa Sheep

**Figure 1:**
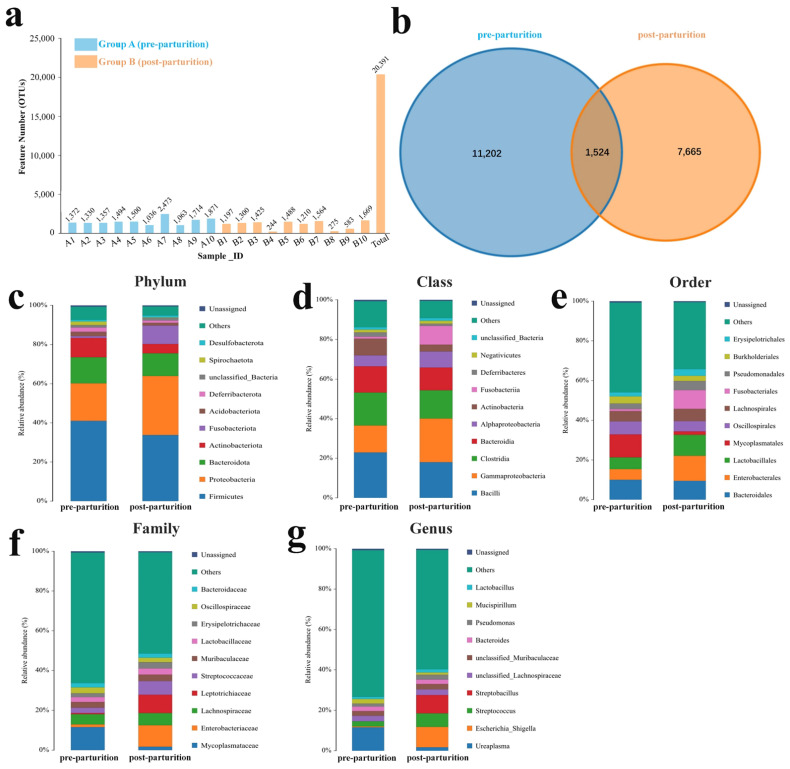
Composition analysis of microbiota in the birth canal of Bangor Sewa Sheep. (**a**) Distribution of OTUS in each sample. (**b**) Venn map. OTUS. Compare the composition of microbiota at (**c**) phylum, (**d**) class, (**e**) order, (**f**) family, and (**g**) genus levels. A1–A10 represent samples from the pre-parturition group, and B1–B10 are samples from the post-parturition group.

The microbiota composition in the birth canal of Bangor Sewa sheep was studied using 16S rRNA sequencing. In the present study, over 54,000 clean reads were generated in each Bangor Sewa sheep sample (Table S1), which were clustered into 20,391 OTUs with 1524 OTUs shared by two groups (Figure 1a,b). The relative abundances of genital tract microbiota in pre- and post-parturition groups were investigated at the phylum, class, order, family, and genus levels. At the phylum level, the most dominant phylum was Firmicutes (41.03% and 33.79%), followed by Proteobacteria (19.19% and 30.18%) and Bacteroidota (13.33% and 11.60%), both in pre- and post-parturition groups (Figure 1c). When compared to the pre-parturition group, the ratios of Firmicutes/Bacteroidota were slightly reduced in the post-parturition group. The dominant classes were Bacilli, Gammaproteobacteria, Clostridia, and Bacteroidia in the pre-parturition group (22.90%, 13.63%, 16.69%, and 13.23%) and the post-parturition group (18.01%, 22.06%, 14.29%, and 11.48%) (Figure 1d). Additionally, the staple orders in the pre-parturition group were Mycoplasmatales (11.58%), Bacteroidales (9.98%), Oscillospirales (6.62%), and Lactobacil-lales (5.91%), and the preponderant orders in the post-parturition group were Enterobacterales (12.62%), Lactobacillales (10.60%), Bacteroidales (9.46%), and Fusobacteriales (9.44%) (Figure 1e). At the family level, the microbiota in the birth canal of Bangor Sewa sheep consists of *Mycoplasmataceae* (11.58%), *Lachnospiraceae* (5.16%), *Oscillospiraceae* (2.93%), *Lactobacillaceae* (2.38%), and others were in the pre-parturition group (Figure 1f). And in the post-parturition group, the microbiota was composed of *Enterobacteriaceae* (10.79%), *Leptotrichiaceae* (9.11%), *Streptococcaceae* (6.91%), *Lachnospiraceae* (6.15%), and so on (Figure 1f). At the genera level, the prime genera were *Ureaplasma* (11.56%), *Streptococcus* (2.55%), and unclassified_*Muribaculaceae* (2.34%) in pre-parturition group and *Escherichia_Shigella* (10.08%), *Streptobacillus* (8.98%), and *Streptococcus* (6.73%) in the post-parturition group (Figure 1g). These data show that the microbiota in the birth canal of Bangor Sewa Sheep differed in composition before and after delivery.

### 3.2. Comparing Microbiota Structure between the Pre- and Post-Parturition of Bangor Sewa Sheep Genital Tracts

**Figure 2:**
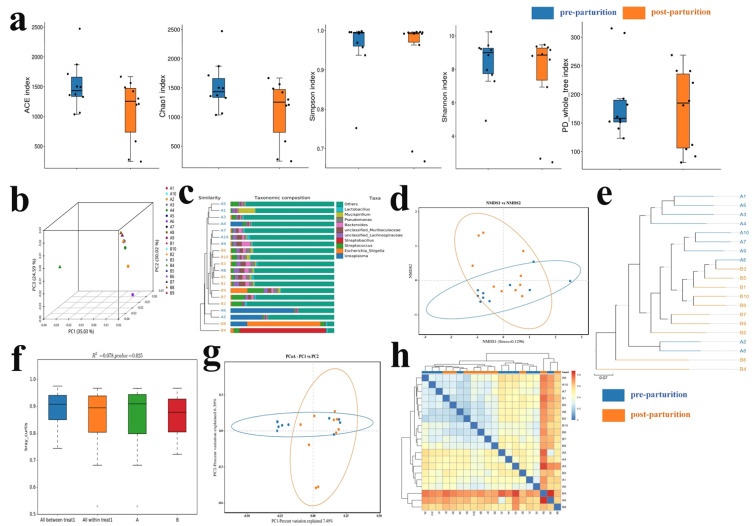
Composition of microbiota structure and diversity of Bangor Sewa sheep in pre- and post-parturition groups. (**a**) Alpha diversity index analysis. Beta diversity was analyzed via PCA (**b**), UPGMA clustering tree (**c**), NMDS (**d**), UPGMA (**e**), PERMANOVA/Anosim (**f**), PCoA (**g**), and Heatmap (**h**). A1–A10 represent samples from the pre-parturition group, and B1–B10 are samples from the post-parturition group. Data are presented as the mean ± SEM (*n* = 10).

To further characterize microbial compositions in Bangor Sewa sheep, we compared the microbiota structure between pre- and post-parturition groups. The alpha diversity index analysis indicated that microbiota in the two groups did not appear to differ significantly; however, microbiota in pre-parturition had slightly higher ACE and Chao 1 indices (Figure 2a and Figure S2, Table S2). Beta diversity analysis showed that although no significant difference in the sample cluster was found, the two sheep groups had a little bit of distinction (Figure 2b–h).

### 3.3. The Remarkable Species between Pre- and Post-Parturition of Bangor Sewa Sheep Genital Tract

**Figure 3:**
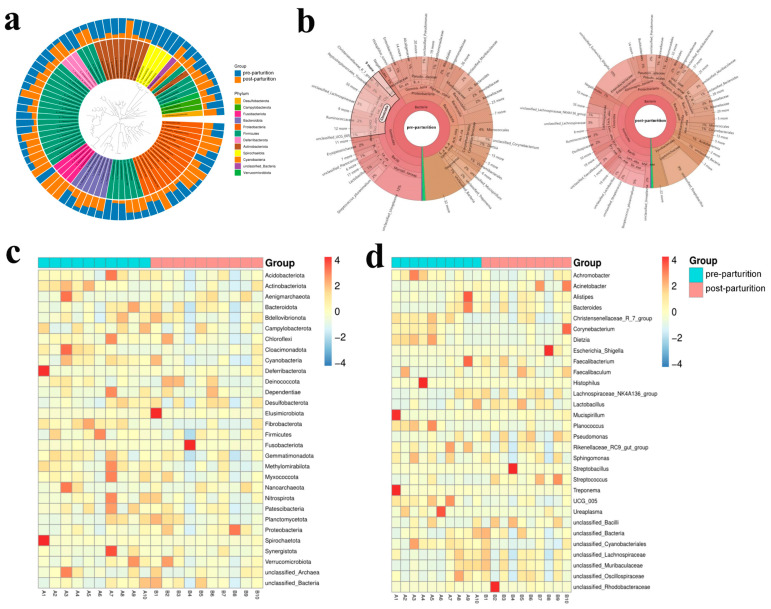
Microbiota comparison of Bangor Sewa sheep performed by the evolutionary tree (**a**), KRONA (**b**), heatmap at phylum, (**c**) and genus (**d**) levels. A1–A10 represents samples from the pre-parturition group and B1–B10 were represents samples from the post-parturition group.

Compared to the pre-parturition group, the microbiota in the post-parturition group had higher levels of Deferribacterota, Firmicutes, Desulfobacterota, Fusobacteriota, Bacteroidota, Proteobacteria, and Verrucomicrobiota while having lower levels of Spirochaetota, Cyanobacteria, and Actinobacteriota at phylum level according to the evolutionary tree analysis (Figure 3a). The KRONA analysis showed that the genus with the highest abundance was unclassified_*Ureaplasma* (12%), followed by unclassified_*Lachnospiraceae* (3%), *Streptococcus_pluranimalium* (2%), *Ruminococcaceae* (2%), *Histophilus_somni* (2%), unclassified_*Muribaculaceae* (2%), *Prevotellaceae* (2%), unclassified_*Mucispirillum* (2%), and unclassified_*Treponema* (2%) in the pre-parturition group, while unclassified_*Escherichia_Shigella* (10%), unclassified_*Streptobacillus* (9%), *Burkholderiales* (3%), unclassified_*Muribaculaceae* (3%), unclassified_*Pseudomonas* (2%), and *Prevotellaceae* (2%) were the dominant genera in the post-parturition group (Figure 3b). Furthermore, the clustering heat map of species abundance indicated that compared with the post-parturition group, the phylum abundance of Actinobacteriota, Campylobacterota, Cloacimonadota, Cyanobacteria, Fibrobacterota, and Spirochaetota were higher, while the levels of Fusobacteriota and Proteobacteria were lower in the pre-parturition group (Figure 3c). At the genera level, the abundance of *Achromobacter*, *Christensenellaceae*_R_7_group, *Corynebacterium*, *Dietzia*, *Histophilus*, *Planococcus*, *Treponema*, UCG_005, and *Ureaplasma* was higher, whereas *Acinetobacter*, *Escherichia_Shigella*, *Faecalibaculum*, *Streptobacillus*, and unclassified_*Rhodobacteraceae* were lower in the pre-parturition group compared with the post-parturition group (Figure 3d).

**Figure 4:**
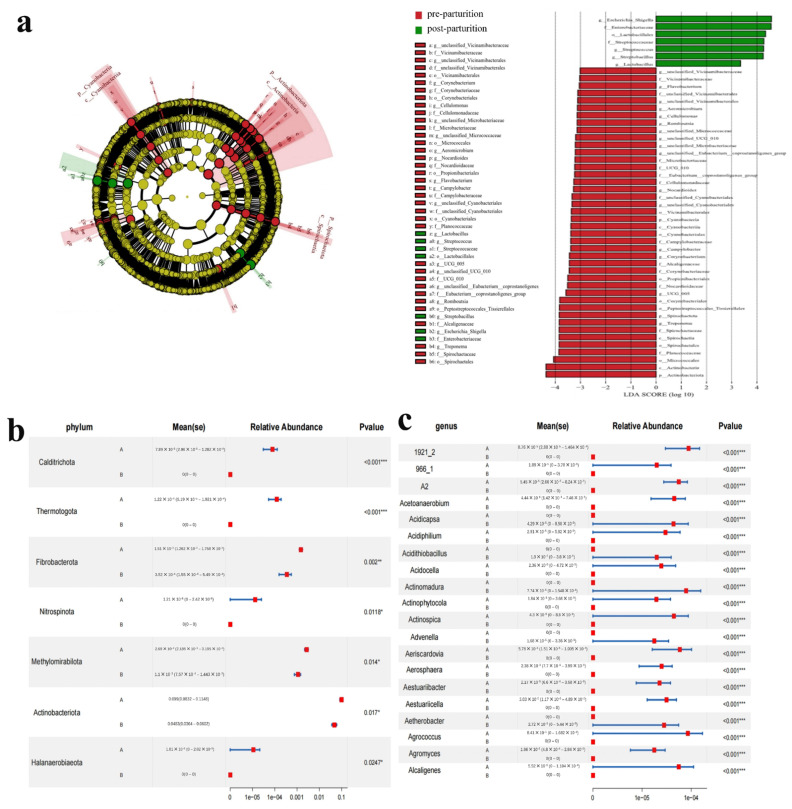
Microbes significantly altered between the two groups of Bangor Sewa sheep are analyzed via LEfSe (**a**) and Metastats at the phylum (**b**) and genus (**c**) levels. A represents the pre-parturition group, and B represents post-parturition group. Significance is presented as * *p* < 0.05; ** *p* < 0.01; *** *p* < 0.001. Data are presented as the mean ± SEM (*n* = 10).

In addition, we also found that, compared to the pre-parturition group, microbiota in the post-parturition group had a higher abundance of g_*Lactobacillus*, g_*Streptococcus*, f_*Streptococcaceae*, o_*Lactobacillales*, g_*Streptobacillus*, g_*Escherichia_Shigella*, and f_*Enterobacteriacea* (Figure 4a). On the contrary, a subset of species had lower abundance, including g_unclassified_*Vicinamibacteraceae*, f_*Vicinamibacteraceae*, g_*unclassified_Vicinamibacterales*, f_*unclassified_Vicinamibacterales*, o_*Vicinamibacterales*, g_*Corynebacterium*, f_*Corynebacteriaceae*, o_*Corynebacteriales*, g_*Cellulomonas*, f_*Cellulomonadaceae*, g_*unclassified_Microbacteriaceae*, f_*Microbacteriaceae*, g_*unclassified_Micrococcaceae*, o_*Micrococcales*, g_*Aeromicrobium*, g_*Nocardioides*, f_*Nocardioidaceae*, o_*Propionibacteriales*, g_*Flavobacterium*, g_*Campylobacter*, f_*Campylobacteraceae*, g_*unclassified_Cyanobacteriales*, f_*unclassified_Cyanobacteriales*, o_*Cyanobacteriales*, f_*Planococcaceae*, g_UCG_005, g_unclassified_UCG_010, f_UCG_010, g_unclassified_*Eubacterium*_*coprostanoligenes*_group, f_*Eubacterium*_*coprostanoligenes_group*, g_*Romboutsia,* o_*Peptostreptococcales_Tissierellales,* f_*Alcaligenaceae*, g_*Treponema*, f_*Spirochaetaceae*, and o_*Spirochaetales* after delivery, according to LEfSe analysis (Figure 4a). Metastats discovered that the phyla of Calditrichota, Thermotogota, Fibrobacterota, Nitrospinota, Methylomirabilota, Actinobacteriota, and Halanaerobiaeota notably decreased after delivery in the birth canal of the Bangor Sewa sheep (Figure 4b). Additionally, the abundance of *Acetoanaerobium*, *Acidiphilium*, *Acidocella*, *Actinophytocola*, *Actinospica*, and other genera markedly declined in the post-parturition group compared to the pre-parturition group, whereas the genera of *Acidicapsa*, *Acidithiobacillus*, *Actinomadura*, *Advenella*, and *Aetherobacter* were elevated after delivery (Figure 4c).

**Figure 5:**
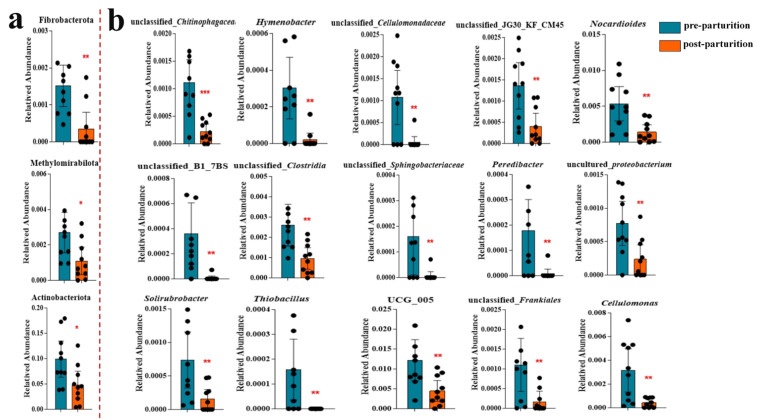
Comparative analysis of the relative abundance of microbiota between the two Bangor Sewa sheep groups at the phylum (**a**) and genus (**b**) levels analyzed via ANOVA. Significance is presented as * *p* < 0.05; ** *p* < 0.01; and *** *p* < 0.001. Data are presented as the mean ± SEM (*n* = 10).

Furthermore, we also discovered that the abundance of Fibrobacterota, Methylomira-bilota, and Actinobacteriota had prominently dropped after delivery compared to that in the earlier group analyzed using ANOVA (Figure 5a). At the genus level, the contents of a large number of bacteria, including unclassified_*Chitinophagaceae*, *Hymenobacter*, unclassified_*Cellulomonadaceae*, *Nocardioides*, *Cellulomonas*, unclassified_*Clostridia*, unclassified_*Sphingobacteriaceae*, *Peredibacter*, uncultured_*proteobacterium*, unclassified_*Frankiales*, and so on, were lower in the post-parturition group relative to the pre-parturition group (Figure 5b). At the same time, the levels of *Streptococcus*, *Facklamia*, *Woeseia*, *Erythrobacter*, and *Bosea* were prominently elevated after delivery in the genital tract of the Bangor Sewa sheep (Figure 5b and Figure S3). Although no remarkable difference was found in *Escherichia_Shigella* between the two groups of Bangor Sewa sheep (Figure S4), network analysis showed that *Mucispirillum* was positively related to the Bangor Sewa sheep microbiota, while *Bacteroides*, unclassified_*Planococcaceae*, unclassified_*Lachnospiraceae*, *Pseudomonas*, *Acinetobacter*, and *Ureaplasma* were negatively related genera (Figure S5). Our results proved that microbial diversity was changed before and after delivery in the genital tract of Bangor Sewa sheep.

### 3.4. Comparing the Microbiota Function between the Two Bangor Sewa Sheep Groups

**Figure 6:**
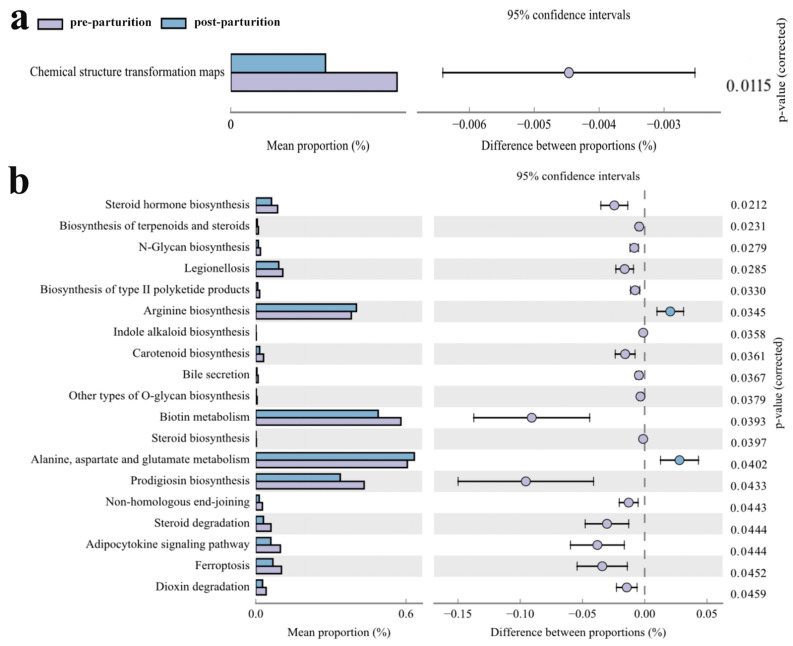
Comparing the microbiota Tax4Fun function between the two Bangor Sewa sheep groups at Level 2 (**a**) and Level 3 (**b**). Data are presented as the mean ± SEM (*n* = 10).

To explore the changes in microbial-related biological functions, we made predictions in a variety of ways. KEGG function predictions found that there were 2, 2, and 23 distinctly different metabolic pathways between two groups at level 1, level 2, and level 3, respectively (Table S3). Although no significantly different functions were discovered between the two groups of Bangor Sewa sheep using BugBase, five obviously different abundant functions were found according to FAPROTAX (Table S4). There was no appreciable difference in the Tax4Fun function at level 1, while the chemical structure transformation map function was noticeably greater at level 2 in the pre-parturition group than that in the post-parturition group (Figure 6a). Furthermore, a series of functions showed obviously different between two groups, indicating that the changed microbes induced multiple functional differences, including steroid hormone biosynthesis, the biosynthesis of terpenoids and steroids, N-glycan biosynthesis, legionellosis, the biosynthesis of type II polyketide products, arginine biosynthesis, indole alkaloid biosynthesis, carotenoid biosynthesis, bile secretion, other types of O-glycan biosynthesis, biotin metabolism, steroid biosynthesis, alanine, aspartate and glutamate metabolism, prodigiosin, non-homologous end-joining, steroid degradation, adipocytokine signaling pathway, ferroptosis, and dioxin degradation (Figure 6b).

## 4. Discussion

This study characterized the dynamic change in the reproductive tract microbiota of Bangor Sewa sheep before and after delivery. Our research showed significant differences in bacterial richness and diversity, including three phyla and 74 genera, between pre- and post-parturition groups. These findings provide valuable insights into the dynamics of the genital tract microbiota during the peripartum period in plateau Bangor Sewa sheep and contribute to our understanding of the mechanisms of physiological changes in this species.

The genital microbiome is made up of several bacterial species. Fungi, viruses, and other microorganisms contribute to the overall microbial ecosystem. The genital microbiome is characterized by a high level of species richness, meaning it contains a wide variety of different microorganisms [24]. A healthy and diverse microbiome is often associated with better vaginal and reproductive health, while dysbiosis, or an imbalance in microbial composition, has been linked to various reproductive tract infections, such as bacterial vaginosis or yeast infections [25,26]. Several factors influence the diversity of the genital microbiome. Host genetics, immune factors, hormonal influences, sexual behavior, antibiotic use, hygiene practices, and reproductive history can all affect the composition and diversity of the genital microbiome [27,28,29]. Additionally, external environmental factors, such as diet and exposure to certain chemicals, might also be important [30]. The composition of the genital microbiome can fluctuate over time due to various factors, including hormonal changes [31], sexual activities [32], menstrual cycle phase [33], and hygiene practices [34]. These fluctuations can lead to changes in the diversity and abundance of different microbial species.

Studies have identified numerous bacterial species in the genital tract, including *Lactobacillus*, *Gardnerella*, *Streptococcus*, *Prevotella*, and others [35]. The diversity of present microorganisms may vary between individuals and even within different parts of the reproductive tract. In healthy animals, the genital microbiome is often dominated by one or a few species of bacteria. The most common dominant bacterial genus is *Lactobacillus*. However, the specific *Lactobacillus* species can vary between individuals. Other bacterial species may also be present but in lower abundances. During pregnancy, the health of the mother and infant may also be affected directly by the birth canal status [36]. We investigated the pre- and post-parturition microbiota of Bangor Sewa sheep since the microbiome balance is a significant determinant of host health.

In cattle and sheep, Firmicutes, Bacteroidetes, and Proteobacteria are the most common phyla in the genital tract [37], and this was consistent with our research. At the genera level, *Ureaplasma*, *Streptococcus*, and *Escherichia_Shigella* were abundant in both the pre-parturition and post-parturition groups. The microbiota of Bangor Sewa sheep showed a considerable transition from pre-parturition to post-parturition. Major variations in the abundance of the three phyla and 74 tax led to the function changes in Bangor Sewa sheep. These alterations might be produced by a variety of causes, including hormone shifts, immunological reactions, and physiological changes in the reproductive organs [38,39], which are worth investigating for further studies.

For the group of marker genera, previous studies examined a higher abundance of unclassified_*Cellulomonadaceae* in herdsmen and pigs on the plateau [40]; *Cellulomonas* in high doses of choline fed to sows [41]; *Fibrobacter* in healthy horses compared with animals with asthma [42]; *Flavobacterium* in healthy rats compared with animals with coronary heart disease [43]; *Eubacterium_ventriosum*_group in healthy people compared with patients with inflammatory bowel disease [44]; and *Acetitomaculum* in healthy Tibetan pigs compared with animals infected with Echinococcus granulosus [45], which may reveal that these genera are species related to the health of host animals. *Dietzia* [46], *Aeromicrobium* [47], and *Parvibacter* [48] are reported as probiotic genera; the lower abundance of those three genera in sheep after calving may demonstrate the sub-healthy status of Bangor Sewa sheep. Previous research found lower abundances of *Nocardioides* in Salmonella-infected pigs [49]; unclassified_*Clostridia* in females with osteoporosis and osteopenia [50]; *Sphingobacteriaceae* in obese people [51]; UCG_005 and RB41 in heat-stressed rabbits [52]; dgA_11_gut_group in rabbits model of Alzheimer disease [53]; unclassified_*Ruminococcaceae* in people with hepatitis B [54]; NK4A214_group in patients with rectovaginal fistula [55]; V9D2013_group in piglets treated with Lipopolysaccharide (LPS) [56]; *Prevotellaceae*_UCG_004 in zearalenone-exposed mice [57]; *Monoglobus* in sleep-deprived mice [58]; *Christensenellaceae*_R_7_group in Alzheimer’s and dementia patients [59]; and *Micromonospora* in LPS-induced inflammation in chicks [60], which indicated that those genera were negatively related to host status. In the current results, the abundance of those genera was significantly changed in post-parturition in the genital tract of Bangor Sewa sheep, which may be closely tied to the changes in the microenvironment, including immune and hormonal factors.

Moreover, *Streptococcus*, *Facklamia*, and *Bosea* were previously reported to be higher in people with an over-richness of bacteria in the intestine [61], type 2 diabetic animals [62], and mice with nonalcoholic fatty liver disease [63], respectively. The higher abundance of those three genera in the post-parturition Bangor Sewa sheep further indicated the changes in the physiological environment of the genital tract. *Escherichia_Shigella*, a related pathogenic and proinflammatory genus [64], showed higher abundance after delivery in Bangor Sewa sheep, which might be a prelude to inflammation. However, research on the involvement of microbes in animal reproduction is relatively restricted, and more study is needed. As microbiota dysbiosis is strongly related to disease status [65], these results might aid in our understanding of the physiological changes that occurred in Bangor Sewa sheep during peripartum and provide a theoretical framework for analyzing the effects of microbial alterations on sheep health.

## 5. Conclusions

In conclusion, we compared the pre-parturition and post-parturition microbiota in Bangor Sewa sheep. The vaginal track’s microbiome revealed different microbial profiles before and after birth. A total of three phyla and 74 genera were observed to have noticeably changed. These results expand our understanding of the microbial composition and changes during peripartum in the genital tract and present a new theoretical basis for improving the health and production of plateau Bangor Sewa sheep.

## Data Availability

All raw sequence data from yak calves were deposited into the NCBI database under the accession number PRJNA985282.

## Supplementary Materials

The following supporting information can be downloaded at: https://www.mdpi.com/article/10.3390/vetsci10080523/s1. Figure S1: The sheep breeding information in China during 2013–2022; Figure S2: The Rarefaction curve, Shannon index curve, Rank abundance curve and Species accumulation curve of Bangor Sewa sheep sequencing data; Figure S3: Obviously changed genu between the two Bangor Sewa sheep groups analyzed by ANOVA; Figure S4: Comparing of the abundance of *Escherichia_Shigella* between the two Bangor Sewa sheep groups; Figure S5: Network analyzing of the bacteria in Bangor Sewa sheep groups; Table S1: Analysis of Bangor Sewa sheep sequencing data; Table S2: Statistical analysis of Alpha diversity indices of Bangor Sewa sheep; Table S3: Comparing the microbiota KEGG function between the two Bangor Sewa sheep groups; Table S4: Comparing the microbiota FAPROTAX function between the two Bangor Sewa sheep groups.
