# Supplementary material for: Comparative Analysis of the Pre-Parturition and Post-Parturition Genital Tract Microbiota in Plateau Bangor Sewa Sheep
Source: Vet Sci. 2023 Aug 14;10(8):523. doi: 10.3390/vetsci10080523 (PMC10459245; doi:10.3390/vetsci10080523)
Supplement: Supplementary file 1 [file vetsci-10-00523-s001.zip › vetsci-2503440-supplementary.pdf]

Supplementary Materials:

Table S1. Analysis of Bangor Sewa sheep sequencing data.

Group	Sample ID	Raw Reads	Clean Reads	Denoised Reads	Merged Reads	Non-chimeric Reads
pre-parturition	A1	80008	79685	76034	70092	64288
	A2	79579	79260	75915	70229	66540
	A3	54685	54463	50392	42458	39560
	A4	80029	79715	75826	68444	63350
	A5	80039	79749	76535	71511	65437
	A6	80290	80007	76965	72049	69869
	A7	79873	79552	71249	56502	49857
	A8	79846	79500	77639	75605	72509
	A9	79944	79638	73129	60180	53488
	A10	79866	79536	75782	68955	63699
post-parturition	B1	80292	79976	77835	74239	71246
	B2	79615	79275	75437	69451	64366
	B3	79992	79627	77368	73891	69906
	B4	80111	79832	77824	75549	71733
	B5	79925	79614	76767	72147	68473
	B6	66461	66173	63712	60614	58494
	B7	79879	79589	74373	65928	60776
	B8	79980	79633	77555	75084	70138
	B9	74558	74277	73243	71341	68868
	B10	79626	79321	75328	68303	62639

Table S2. Statistical analysis of Alpha diversity indices of Bangor Sewa sheep.

Group	Sample	Feature	ACE	Chao1	Simpson	Shannon	PD_whole_tree
pre-parturition	A1	1372	1372.3307	1372.0213	0.9689	7.9589	140.4025
	A2	1330	1330.1819	1330.0000	0.9372	7.2881	152.6300
	A3	1357	1357.1915	1357.0000	0.9958	9.2154	182.3420
	A4	1494	1494.5995	1494.0435	0.9575	7.6686	315.3979
	A5	1500	1500.3697	1500.0179	0.9924	8.8508	307.5412
	A6	1036	1036.1727	1036.0000	0.7522	4.9204	151.4822
	A7	2473	2473.1993	2473.0000	0.9983	10.2448	161.001
	A8	1063	1063.0000	1063.0000	0.9958	9.1543	192.5915
	A9	1714	1714.0000	1714.0000	0.9958	9.2756	123.3023
	A10	1871	1871.1589	1871.0000	0.9956	9.4306	155.5691
post-parturition	B1	1197	1197.2293	1197.0000	0.9963	9.3077	240.9322
	B2	1300	1300.6455	1300.0429	0.9918	8.5814	268.8573
	B3	1425	1425.4083	1425.0476	0.9963	9.4703	111.7690
	B4	244	244.5821	244.0345	0.6677	2.4373	104.5365
	B5	1488	1488.2008	1488.0000	0.9949	9.3637	91.8396
	B6	1210	1210.0000	1210.0000	0.9952	9.1576	241.3505
	B7	1564	1564.0000	1564.0000	0.9921	8.9000	220.4017
	B8	275	275.4582	275.0357	0.6928	2.6515	181.1489
	B9	583	583.0000	583.0000	0.9632	6.9359	81.0445
	B10	1669	1669.0000	1669.0000	0.992	8.8032	268.8573

Table S3. Comparing the microbiota KEGG function between the two Bangor Sewa sheep groups. Data are presented as the mean ± SEM (n = 10).

KEGG level	Function	A (Relative frequency, %)	A (Relative frequency, %)	P value
Level 1	Human Diseases	2.6562±0.1060	2.9458±0.3045	0.0206
	Environmental Information Processing	6.7557±0.2027	7.3421±0.7478	0.0458
Level 2	Transport and catabolism	0.2740±0.0117	0.2405±0.0391	0.0325
	Energy metabolism	4.1545±0.0496	4.0939±0.0647	0.0396
Level 3	mRNA surveillance pathway	0.0001±0.0000	0.0000±0.0000	0.0009
	Adipocytokine signaling pathway	0.0773±0.0080	0.0599±0.0135	0.0048
	Basal transcription factors	0.0044±0.0023	0.0016±0.0012	0.0055
	Steroid biosynthesis	0.0029±0.0011	0.0014±0.0009	0.0055
	Fatty acid elongation	0.0004±0.0002	0.0001±0.0001	0.0057
	Renin-angiotensin system	0.0136±0.0025	0.0084±0.0041	0.0059
	Steroid degradation	0.0055±0.0028	0.0022±0.0014	0.0081
	cAMP signaling pathway	0.0035±0.0020	0.0013±0.0010	0.0091
	Non-homologous end-joining	0.0213±0.0067	0.0122±0.0066	0.0095
	Calcium signaling pathway	0.0002±0.0001	0.0001±0.0001	0.0107
	Stilbenoid, diarylheptanoid and gingerol biosynthesis	0.0067±0.0017	0.0039±0.0025	0.0113
	Peroxisome	0.2116±0.0207	0.1841±0.0210	0.0118
	Longevity regulating pathway - multiple species	0.1145±0.0118	0.1009±0.0103	0.0184
	Citrate cycle (TCA cycle)	0.6575±0.0367	0.5871±0.0716	0.0205
	MAPK signaling pathway - yeast	0.0232±0.0068	0.0157±0.0059	0.0222
	Hematopoietic cell lineage	0.0002±0.0001	0.0001±0.0001	0.0250
	Nonribosomal peptide structures	0.0142±0.0023	0.0108±0.0034	0.0264
	PPAR signaling pathway	0.1747±0.0167	0.1502±0.0269	0.0347
	Sesquiterpenoid and triterpenoid biosynthesis	0.0036±0.0006	0.0025±0.0013	0.0412
	Systemic lupus erythematosus	0.0006±0.0003	0.0003±0.0002	0.0458
	Bacterial secretion system	0.6259±0.0360	0.6912±0.0811	0.0466

Table S4. Comparing the microbiota FAPROTAX function between the two Bangor Sewa sheep groups. Data are presented as the mean ± SEM (n = 10).

Function	A (Relative frequency, %)	A (Relative frequency, %)	P value
dark_sulfide_oxidation	0.0231±0.0189	0.0000±0.0000	0.0052
cellulolysis	0.1744±0.0961	0.0656±0.0624	0.0120
cyanobacteria	1.1932±0.4300	0.6047±0.5570	0.0226
oxygenic_photoautotrophy	1.1932±0.4300	0.6047±0.5570	0.0226
reductive_acetogenesis	0.0187±0.0168	0.0039±0.0118	0.0471

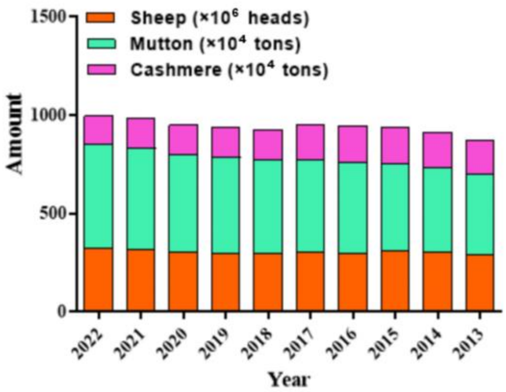

Figure S1. The sheep breeding information in China during 2013-2022.

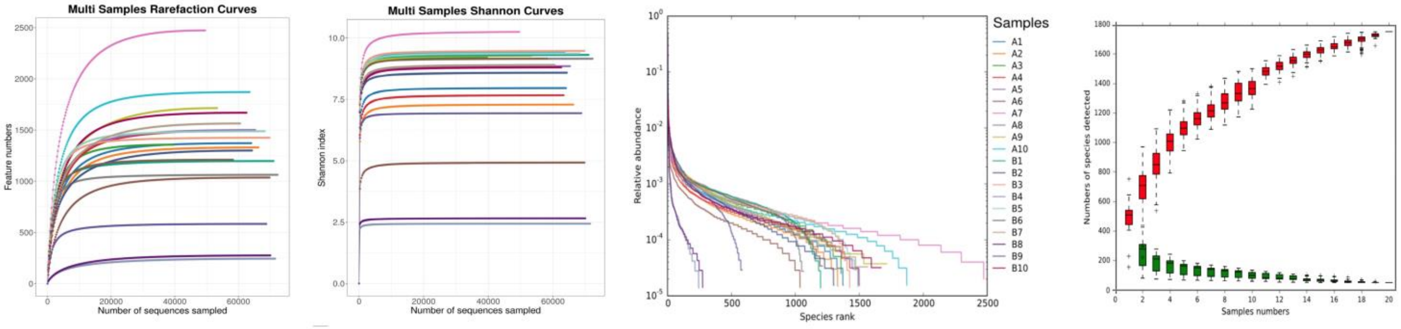

Figure S2. The Rarefaction curve, Shannon index curve, Rank abundance curve and Species accumulation curve of Bangor Sewa sheep sequencing data. A1-A10 represented samples from pre-parturition group, and B1-B10 were samples from the post-parturition group.

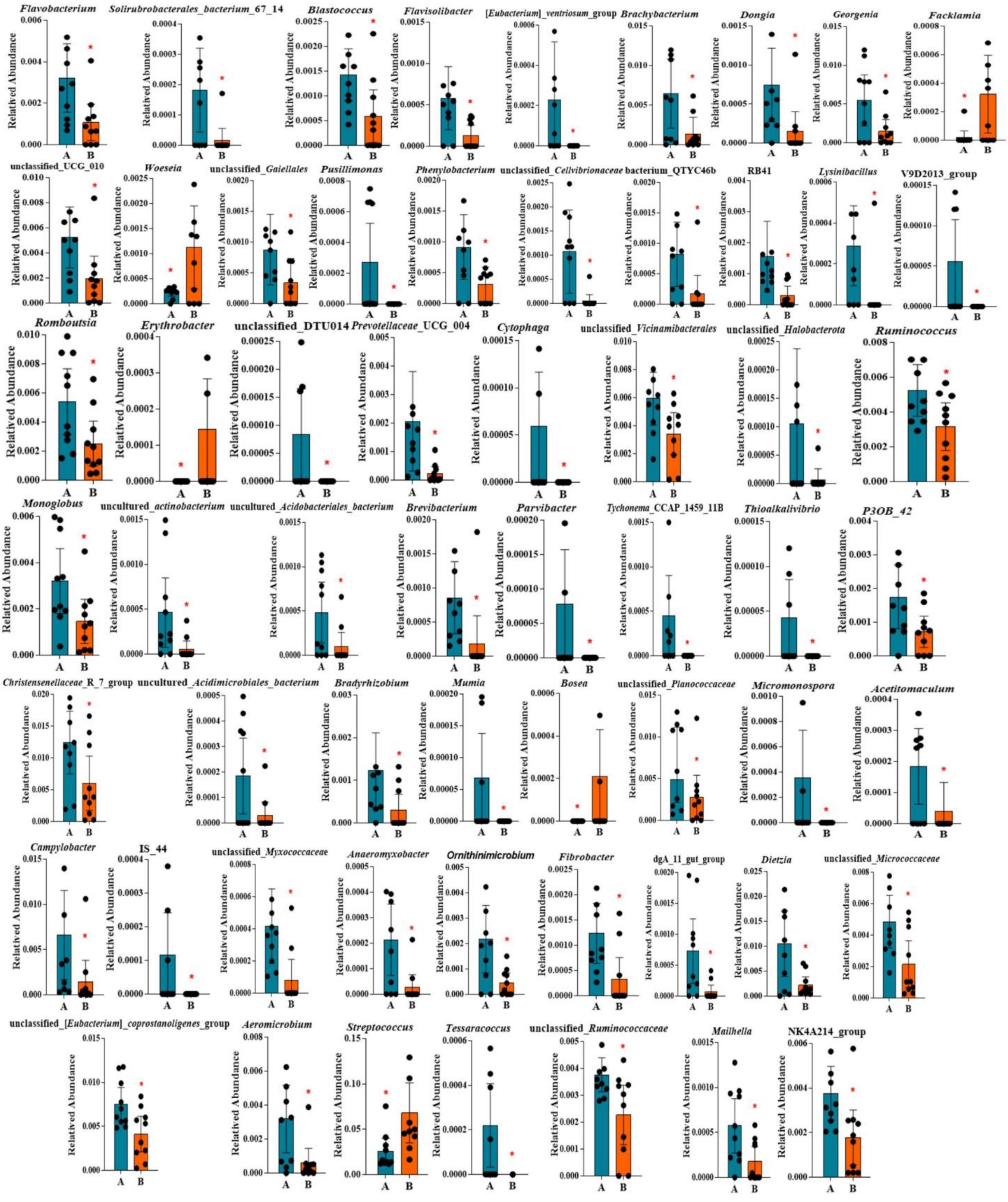

Figure S3. Obviously changed genus between the two Bangor Sewa sheep groups analyzed by ANOVA. A represented pre-parturition group, and B represented post-parturition group. Significance is presented as *p < 0.05. Data are presented as the mean ± SEM (n = 10).

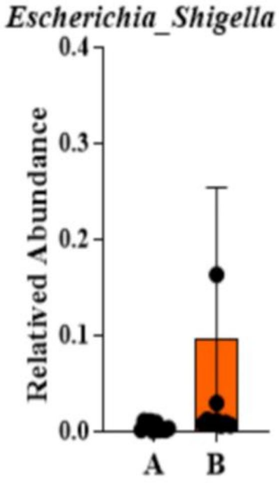

Figure S4. Comparing of the abundance of *Escherichia_Shigella* between the two Bangor Sewa sheep groups. A represented pre-parturition group, and B represented post-parturition group. Significance is presented as *p < 0.05. Data are presented as the mean ± SEM (n = 10).

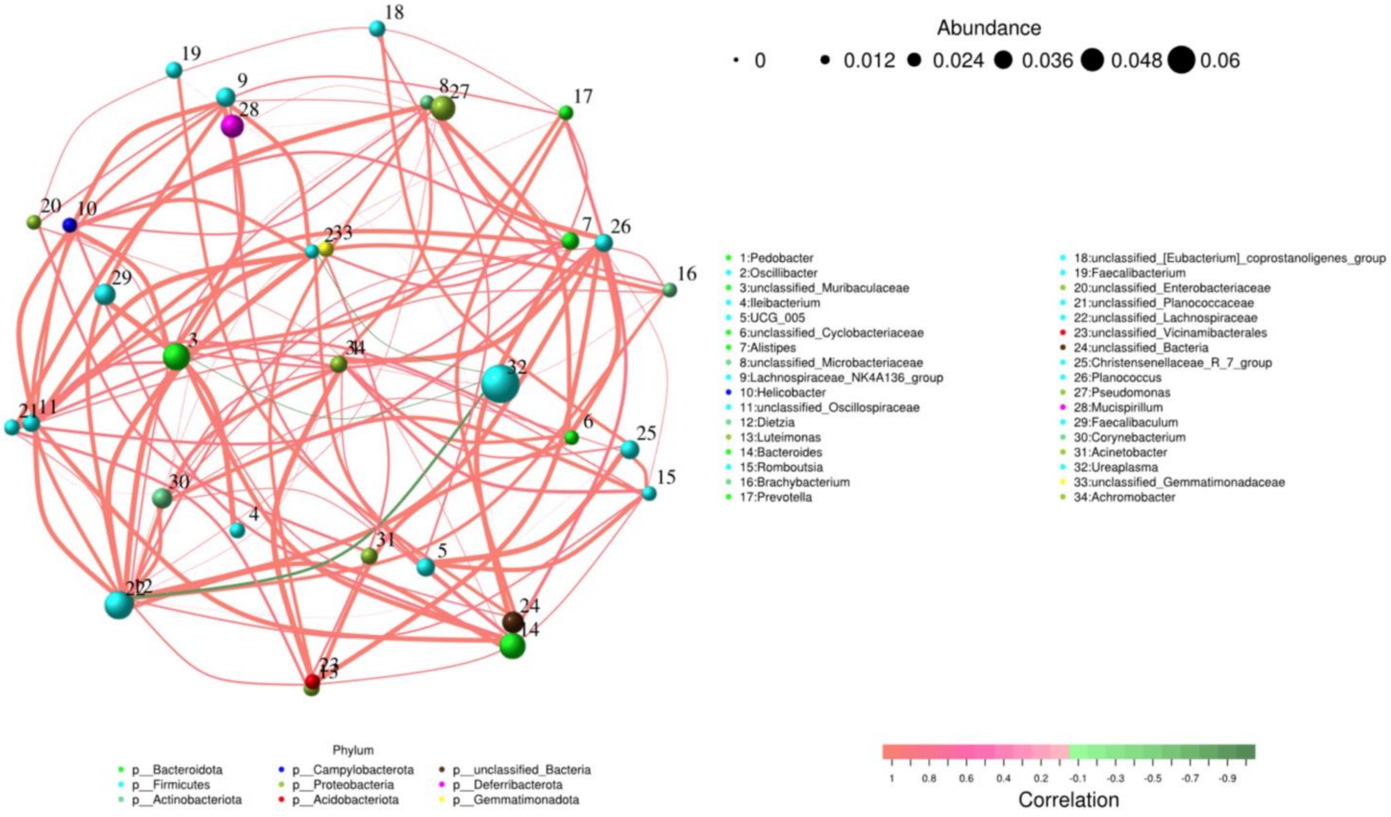

Figure S5. Network analyzing of the bacteria in Bangor Sewa sheep groups.
